# How Iwate Prefecture in Japan maintained a low COVID-19 infection rate

**DOI:** 10.5365/wpsar.2021.12.4.859

**Published:** 2021-10-27

**Authors:** Shuko Takahashi, Ichiro Kawachi

**Affiliations:** a Iwate Prefecture Government, Morioka, Iwate, Japan.; b Division of Medical Education, Iwate Medical University, Iwate, Japan.; c Harvard T.H. Chan School of Public Health, Boston, Massachusetts, United States of America.

The first case of coronavirus disease 2019 (COVID-19) in Japan was confirmed on 16 January 2020. The first wave of cases peaked on 10 April 2020 (*n* = 710) and the second on 7 August 2020 (*n* = 1595). Iwate Prefecture in north-eastern Japan was the last prefecture to confirm a case of COVID-19, on 29 July 2020, 110 days after all other prefectures had confirmed cases. No cases were reported during the first wave. (*1*) As of 21 September 2021, there had been 3469 cases (282.8/100 000 population) and 52 deaths (1.50% fatality rate) in Iwate and 1.7 million cases (1333.2/100 000 population) and 17 294 deaths (1.03% fatality rate) in Japan overall. This article discusses possible reasons for the low number of COVID-19 cases in Iwate.

## Geographical characteristics and population movement

Iwate Prefecture is 500 km from Tokyo and is bordered by mountains to the west and the sea to the east. It has a low population density (83.8 persons/km^2^). Population movement into and within Iwate decreased after the initial COVID-19 cases were reported in Japan.After a national state of emergency was declared on 16 April 2020, the transient population of Morioka City, the capital of Iwate, decreased by 30–60%. (*2*) During the national Golden Week holiday in 2020, held at the end of April, for example, travel on trains to major train stations in Iwate was 70–80% lower than in 2019. (*3*) A survey showed that two thirds of Iwate residents did not want contact with people from other prefectures, (*4*) and people from other prefectures avoided going to Iwate to avoid discrimination. Thus, geographical barriers and decreased movement into Iwate may have contributed to the low transmission.

Miyagi Prefecture neighbours Iwate to the south. Although its historical, demographic, socioeconomic and cultural characteristics are similar to those of Iwate, it had 149 notifications of COVID-19 as of 28 July 2020, while Iwate had none. Miyagi Prefecture is closer to Tokyo, at 300 km, and is also the largest prefecture in the Tohoku region in terms of population and economy. Miyagi Prefecture also had to take in COVID-19 patients who were infected on board the Diamond Princess cruise ship without adequate preparation. (*5*) These factors may have contributed to a higher rate of contact between people and more cases.

## Lessons learnt from responding to the 2011 Great East Japan Earthquake and Tsunami

Countermeasures for infectious diseases were established in Iwate to respond to the 2011 Great East Japan Earthquake and Tsunami. These included use of infection control assistance teams for daily surveillance, training in hand hygiene and providing information on infection control. (*6*) The teams were used in the early response to COVID-19 in Iwate and provided advice and information to decision-makers for infection control.

## Countermeasures adopted by the Iwate prefectural government

The Iwate prefectural government took appropriate local actions at each stage of the COVID-19 pandemic. It established a countermeasure headquarters headed by the governor in February 2020, with the first phase of countermeasures beginning on 23 April when a state of emergency was declared. Although there were no local cases during this phase, the strategy was to limit the risk of transmission by physical distancing. Businesses stayed open, but the government requested people to avoid “unnecessary and non-urgent” outings. (*7*) Schools were closed from 2 to 25 March and from 29 April to 6 May. Staff were recruited for disaster medical assistance teams to coordinate the work of hospitals. Testing and treatment centres and support systems, such as call centres for travellers, were quickly established.

During the second phase, from after the state of emergency in Iwate was lifted on 14 May to 7 June, the prefectural government continued to prevent transmission while maintaining the local economy. Although the national government established restrictions on large-scale events, in Iwate, which had still not reported a COVID-19 case, all schools and businesses (including bars, night clubs and restaurants) remained open, except between 29 April and 6 May, when all recreational facilities, night clubs or establishments that served food and beverages were closed. The prefectural government requested residents not to travel between prefectures, but this request was relaxed on 1 June.

The goal during the third phase was to provide information about the current situation and establish plans for when the first confirmed COVID-19 case occurred. As the period with no confirmed cases in Iwate became longer, residents feared becoming the first case. The prefectural government promised to provide sufficient contact tracing and isolation and publicly appealed that no blame be placed on cases.

Strong leadership throughout the response included clear, consistent messaging by government officials about preventive measures, such as avoiding the “three Cs” (confined spaces with poor ventilation, gathering in crowded areas and close contact with others), frequent hand-washing and physical distancing. (*7*)

The Iwate prefectural government also provided direct support to businesses affected by the COVID-19 restrictions. (*8*) After declaration of the first national state of emergency in late April 2020, many companies experienced financial difficulties; however, as of late July 2020, only two companies in Iwate had closed due to the pandemic. The unemployment rate in Iwate hardly changed (2.1% in 2019 vs 2.4% in 2020). (*9*)

## Cultural characteristics of Iwate residents

The Japanese custom of physical distancing during greetings is often cited as a factor in preventing transmission of infectious diseases. (*10*) Another characteristic of Iwate residents, which may also prevent transmission of respiratory infections, is that they do not raise their voices during conversation. Widespread awareness of being the last prefecture in Japan without a confirmed case of COVID-19 might also have led to further effort to avoid infection.

## Limited testing early in the response

**Figure 1:**
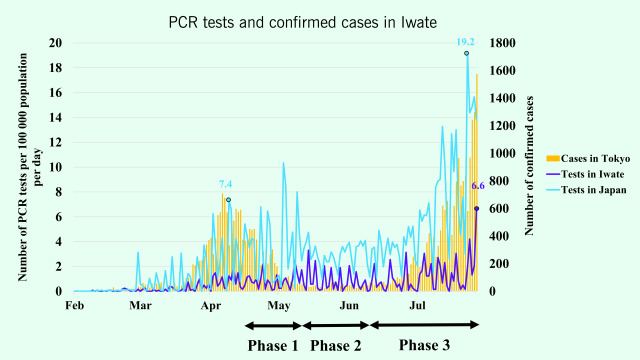
Number of PCR tests conducted and COVID-19 cases confirmed in Iwate and Japan, February–July 2020^a^

Another possible reason for the low number of COVID-19 cases in Iwate is limited testing, as polymerase chain reaction (PCR) tests were initially used only for patients with symptoms of pneumonia. (*11*) As of 28 July 2020, 1438 diagnostic tests had been conducted in Iwate, a rate of 118.4 per 100 000 population, as compared with 515.7 per 100 000 in Japan overall and 1297.7 per 100 000 in Tokyo (**Fig. 1**). (*12*-*14*) Therefore, asymptomatic cases of COVID-19 might have been missed, in particular among younger people. (*15*) As resources for testing increased in Japan, tests were conducted not only for symptomatic patients but also for asymptomatic suspected cases. (*16*) By July 2020, Japan had acquired sufficient testing capacity. Therefore, if cases of infection had been missed due to lack of testing or undetected asymptomatic cases, there should have been a large increase in the number of cases of COVID-19 once testing was increased. (*17*) This was not the case.

[**insert** Figure 1]

This article summarizes the characteristics of Iwate Prefecture, its population and local government actions that may have contributed to the delay in cases of COVID-19 infection. The extra time allowed the local government to strengthen health-care capabilities and raise residents’ level of awareness. These countermeasures might have contributed to the smaller number of reported COVID-19 cases in Iwate, which has continued into the second year of the pandemic.

## References

[1] Information on the Coronavirus (COVID-19) - Iwate Prefecture. Iwate: Prefectural Government; 2021. Available from: https://www.pref.iwate.jp/kyouikubunka/kokusai/1006971/1027622/1027623.html, accessed 23 September 2021.

[2] Comparison of a transient population in the central commercial areas in Morioka City during Golden Week periods between in 2019 and 2020: Iwate: Prefectural Government; 2021. Available from: https://www.pref.iwate.jp/_res/projects/default_project/_page_/001/028/231/20200526_011.pdf, accessed 23 September 2021.

[3] Comparison of population density across consecutive holidays in May in major stations in Iwate. Iwate: Prefectural Government; 2021. Available from: https://www.pref.iwate.jp/_res/projects/default_project/_page_/001/028/231/20200515_004.pdf, accessed 23 September 2021.

[4] Perceptions and behaviours towards COVID-19 (in Japanese). Tokyo: Neo Marketing; 2020. Available from: https://neo-m.jp/investigation/2516/, accessed 23 September 2021.

[5] Mizumoto K, Kagaya K, Zarebski A, Chowell G. Estimating the asymptomatic proportion of coronavirus disease 2019 (COVID-19) cases on board the Diamond Princess cruise ship, Yokohama, Japan, 2020. Euro Surveill. 2020 03;25(10):2000180. 10.2807/1560-7917.ES.2020.25.10.2000180 32183930 PMC7078829

[6] Nohara M. Impact of the Great East Japan Earthquake and tsunami on health, medical care and public health systems in Iwate Prefecture, Japan, 2011. Western Pac Surveill Response J. 2012 12 23;2(4):24–30. 10.5365/WPSAR.2011.2.4.002 23908898 PMC3729067

[7] Tasso T. A message from the Governor of Iwate about COVID-19 (April 3). Iwate: Prefectural Government; 2020. [cited 2021 September 23]. Available from: Available from https://www.pref.iwate.jp/kyouikubunka/kokusai/1006971/1027622/1046827/1028829.html

[8] Tasso T. A message from the Governor of Iwate about COVID-19 (23 April). Iwate: Prefectural Government; 2020. [cited 2021 September 23]. Available from: Available from https://www.pref.iwate.jp/kyouikubunka/kokusai/1006971/1027622/1046827/1029410.html

[9] Summary of labour force survey from 2015 to 2020 (in Japanese). Tokyo: Statistics Bureau, Ministry of Internal Affairs and Communications; 2021. Available from: https://www.stat.go.jp/data/roudou/pref/zuhyou/lty.xlsx, accessed 23 September 2021.

[10] Mahbub M, Khan M, Yamaguchi N, Hase R, Harada N, Tanabe T. Japan’s public health and culture, and the ongoing fight against COVID-19. J Adv Biotechnol Exp Ther. 2020;3(4):42–8. 10.5455/jabet.2020.d155

[11] Legido-Quigley H, Asgari N, Teo YY, Leung GM, Oshitani H, Fukuda K, et al. Are high-performing health systems resilient against the COVID-19 epidemic? Lancet. 2020 03 14;395(10227):848–50. 10.1016/S0140-6736(20)30551-1 32151326 PMC7124523

[12] Information on the coronavirus (COVID-19) (in Japanese). Iwate: Iwate Prefecture; 2021. Available from: https://www.pref.iwate.jp/kurashikankyou/iryou/covid19/index.html, accessed 23 September 2021.

[13] Confirmed cases of the coronavirus (COVID-19) in Japan (in Japanese. Tokyo: Ministry of Health, Labour and Welfare; 2021. Available from: https://www.mhlw.go.jp/stf/covid-19/kokunainohasseijoukyou.html, accessed 23 September 2021.

[14] Updates on COVID-19 in Tokyo. Tokyo: Tokyo Metropolitan Government; 2021. Available from: https://stopcovid19.metro.tokyo.lg.jp/en, accessed 23 September 2021.

[15] Davies NG, Klepac P, Liu Y, Prem K, Jit M, Eggo RM; CMMID COVID-19 working group. Age-dependent effects in the transmission and control of COVID-19 epidemics. Nat Med. 2020 08;26(8):1205–11. 10.1038/s41591-020-0962-9 32546824

[16] Tests for novel coronavirus disease (in Japanese). Tokyo: Ministry of Health, Labour and Welfare; 2021. Available from: https://www.mhlw.go.jp/stf/seisakunitsuite/bunya/0000121431_00132.html?fbclid=IwAR0H7i3LN-TiC0USWlk31qvF7QFc1x_ukmis33CyENp4Q8aeK62pnRCvLpQ, accessed 23 September 2021.

[17] Iwasaki A, Grubaugh ND. Why does Japan have so few cases of COVID-19? EMBO Mol Med. 2020 05 8;12(5):e12481. 10.15252/emmm.202012481 32275804 PMC7207161

